# Editorial: Why the exact frequencies in our brains matter: Perspectives from electrophysiology and brain stimulation

**DOI:** 10.3389/fnsys.2022.1121438

**Published:** 2023-01-04

**Authors:** Avgis Hadjipapas, Charalambos C. Charalambous, Mark J. Roberts

**Affiliations:** ^1^Department of Basic and Clinical Sciences, University of Nicosia Medical School, Nicosia, Cyprus; ^2^Center of Neuroscience and Integrative Brain Research (CENIBRE), University of Nicosia, Nicosia, Cyprus; ^3^Department of Cognitive Neuroscience, Faculty of Psychology and Neuroscience, Maastricht University, Maastricht, Netherlands

**Keywords:** synchronization, frequency, brain stimulation, neuronal oscillations, detuning, electrophysiology

Oscillations are a ubiquitous feature of brain activity and have been studied for nearly a century (Buzsáki et al., 2013; Singer, 2018) yet, the role of their most central characteristic, namely their preferred frequency remains elusive. A commonly supported view is that preferred oscillation frequency should not differ between networks to allow synchronization (Burns et al., 2011; Ray and Maunsell, 2015). Thus, frequency variation has been generally viewed as detrimental. In this special topic we explore an alternative view, that frequency variability allows useful flexibility that may be functionally exploited.

Lowet et al. argue that the preferred oscillation frequency is diverse across the brain and varies flexibly with sensory and cognitive variables. According to synchronization theory, frequency variation between oscillators (so called *detuning*) is a key parameter which, in an antagonistic interplay with coupling strength between the oscillators, controls synchronization (Pikovsky et al., 2002). The relationship between synchronization and these two parameters is described by the Arnold Tongue. The authors highlight that stable detuning may play an important role in the brain by keeping synchronization sufficiently local, supporting functional segregation and avoiding pathological over-synchronization. Detuning can also result in the emergence of non-zero phase lags between networks even when the networks do not have structural delays. These phase lags, determine whether a network is a “driver” or a “follower” (Lowet et al., 2017). In networks that display a systematic frequency gradient, these phase lags combine into traveling waves from high-frequency nodes to low frequency nodes. Since preferred frequencies of networks depend on sensory and cognitive variables, the brain may exploit detuning and the resultant stable phase lags to flexibly shape information flow in behaviorally relevant networks. Thus, detuning should not be seen as detrimental but on the contrary, it can be functionally beneficial to determine the structure of information flow moment-to-moment.

Evers et al., show that synchronization theory, applied to networks in early visual cortex, can have direct consequences for visual function. They start from the premise that preferred oscillation frequency is input (stimulus)-dependent (Roberts et al., 2013) and thus interconnected neighboring cortical areas stimulated by stimuli with varying visual contrast will interact as coupled oscillators in the presence of detuning (Lowet et al., 2015, 2017). They build a computational model of coupled oscillators, whose preferred frequencies are governed by the stimuli in their receptive fields. In simulations mimicking contextual modulation, where there is a target and flanker stimulus, they show that whether the target network is suppressed or facilitated depends on the detuning between target and flanker oscillators, which in turn is governed by visual contrast differences. Their results are consistent with relevant neurophysiological data. They thus extend previous results which postulated such function for local perceptual grouping (Lowet et al., 2015).

Detuning is also important when brain networks are externally periodically stimulated, which represents synchronization by an external force, also known as *entrainment* (Pikovsky et al., 2002). Transcranial alternating current stimulation (tACS) can modulate ongoing brain oscillations by stimulating cortical networks at specific frequencies close to their preferred frequency with consequent neural or behavioral effects; tACS thus can provide a causal means of testing oscillation function. Vogeti et al. outline the evidence for the two main mechanisms believed to underlie tACS effects—entrainment and spike time dependent plasticity (STDP). tACS effects can be divided into “online” effects (effects that occur during stimulation) and “offline” effects' (effects lasting beyond the stimulation). Often “online” effects are related to modulation of behavior across different phases of the stimulation frequency, whereas “offline” effects are post-stimulation neurophysiological effects such as modulations of the oscillation power or coherence. To establish entrainment directly, “online” electrophysiological data need to be examined, which is however hampered by the stimulation artifact. Yet, one can observe “online” behavioral effects of stimulation, which are often attributed to entrainment. On the contrary, “offline” neurophysiological effects may be better explained by SDTP, as in this case, entrainment is not required. Nevertheless, absence of entrainment is not direct evidence for STDP. Vogeti et al. conclude by highlighting an important gap in the literature, namely the paucity of studies that systematically concurrently assess both “online” and “offline” effects, which would be important to disentangle the contribution of entrainment and STDP in tACS effects.

Frequency-specific periodic brain stimulation may have especially beneficial effects in specific patient populations, where a pathophysiological dysfunction of a specific oscillation is hypothesized. Traikapi and Konstantinou review the evidence for gamma frequency stimulation as a potential therapeutic tool in Alzheimer's disease (AD). This approach is based on the notion that gamma-frequency stimulation may alleviate gamma deficiency by entraining these oscillations. A novel approach of applying frequency-specific sensory stimulation in mice models of AD has been shown to ameliorate key AD pathology (Adaikkan et al., 2019). Some early studies in humans provide the first evidence in humans, that gamma sensory stimulation may be feasible and beneficial (He et al., 2021; Chan et al., 2022). However, while studies in mice have shown that the effects are frequency-specific, the same remains to be determined in humans.

Charalambous and Hadjipapas explore the evidence for frequency-specificity of the motor-descending drive during walking, a crucial motor task, which is commonly impaired after stroke. The notion is that if a clear frequency-specificity of the two main descending motor tracts (corticospinal tract, CST and corticoreticulospinal tract, CReST) during walking can be established, then targeted therapy by frequency-specific stimulation of the descending drive (e.g., tACS), may represent a potentially fruitful post-stroke neurorehabilitation strategy. While beta-frequency specificity is well-documented for CST, more studies both in animal models and humans are needed to support alpha frequency specificity of CReST. In addition, there is evidence that tACS applied during walking can modulate both beta-oscillations and behavior, which could prove beneficial after stroke. However, tACS studies thus far did not stimulate at specifically at alpha or beta frequencies, which is an important gap in the literature. Overall, carefully characterizing frequency-specific oscillations in neural and muscular signals during walking seems to be a fruitful approach.

While much is known about the role of oscillation amplitude, in this special topic we show that the precise oscillation frequency is also very relevant. It determines detuning either between networks or between a network and an external stimulus and plays a key role in synchronization. We show that the brain may exploit detuning functionally and that the precise frequency of external stimulation, either sensory or electrical /magnetic, may boost cognition and behavior as well as determine the success in treating brain pathologies.

## Author contributions

AH drafted the manuscript. All authors conceptualized the editorial. All authors read, edited, and approved the submitted version.
